# The COPD-Associated Polymorphism Impairs the CFTR Function to Suppress Excessive IL-8 Production upon Environmental Pathogen Exposure

**DOI:** 10.3390/ijms24032305

**Published:** 2023-01-24

**Authors:** Daichi Hinata, Ryosuke Fukuda, Tsukasa Okiyoneda

**Affiliations:** Department of Biomedical Sciences, School of Biological and Environmental Sciences, Kwansei Gakuin University, Hyogo 669-1330, Japan

**Keywords:** CFTR, COPD, polymorphism, cystic fibrosis, IL-8, Trikafta, CFTR modulator

## Abstract

COPD is a lifestyle-related disease resulting from irreversible damage to respiratory tissues mostly due to chronic exposure to environmental pollutants, including cigarette smoke. Environmental pathogens and pollutants induce the acquired dysfunction of the CFTR Cl^−^ channel, which is invoked in COPD. Despite the increased incidence of CFTR polymorphism R75Q or M470V in COPD patients, the mechanism of how the CFTR variant affects COPD pathogenesis remains unclear. Here, we investigated the impact of CFTR polymorphisms (R75Q, M470V) on the CFTR function in airway epithelial cell models. While wild-type (WT) CFTR suppressed the proinflammatory cytokine production induced by COPD-related pathogens including pyocyanin (PYO), R75Q- or M470V-CFTR failed. Mechanistically, the R75Q- or M470V-CFTR fractional PM activity (FPMA) was significantly lower than WT-CFTR in the presence of PYO. Notably, the CF drug Trikafta corrected the PM expression of R75Q- or M470V-CFTR even upon PYO exposure and consequently suppressed the excessive IL-8 production. These results suggest that R75Q or M470V polymorphism impairs the CFTR function to suppress the excessive proinflammatory response to environmental pathogens associated with COPD. Moreover, Trikafta may be useful to prevent the COPD pathogenesis associated with acquired CFTR dysfunction.

## 1. Introduction

Chronic obstructive pulmonary disease (COPD) is a lifestyle-related syndrome associated with chronic airway obstruction that is caused by chronic exposure to pollutants, especially cigarette smoke (CS) [1]. It is currently estimated that 80 million people worldwide suffer from COPD, making it the third leading cause of death [2]. Pharmacological therapy for COPD is limited to symptomatic treatments [1], and thus, there is a need for the development of effective therapies. In COPD, sustained airway inflammation and chronic bronchial infections lead to worsening airway obstruction, progressive dyspnea, and acute respiratory exacerbations [1]. Each of these clinical manifestations is also a cardinal feature of cystic fibrosis (CF), raising the possibility that common pathogenic mechanisms exist in CF and COPD [3].

CF is a monogenic disease caused by mutations of the gene encoding the CF transmembrane conductance regulator (CFTR) [4]. CFTR is a cAMP-regulated Cl^−^ channel expressed on the apical plasma membrane (PM) of epithelial cells, including the airway. Since CFTR also negatively regulates the epithelial sodium channel (ENaC), the loss of functional CFTR expression causes a decreased Cl^−^ export and excessive Na^+^ absorption, leading to airway surface liquid (ASL) dehydration [5,6]. Therefore, CFTR plays an important role in airway clearance to protect the epithelium from infection [6]. Previous studies have shown that individuals with COPD not only decrease their CFTR function in respiratory epithelial cells but also show significant correlations between clinical manifestations of COPD and the level of CFTR suppression in their lower respiratory tract [7,8,9,10]. Moreover, CS exposure, which is the most cause of COPD, promotes CFTR internalization and channel inactivation [11,12,13,14]. Thus, the acquired CFTR dysfunction caused by environmental pathogens and/or pollutants has been proposed to contribute to the development of COPD [8,15,16,17].

CS contains about 4,000 different components, including sources of oxidants, such as cadmium (Cd) [18]. Cd is a highly toxic heavy metal that produces reactive oxygen species (ROS) by disrupting the electron transfer chain in mitochondria [19]. Each cigarette contains 1–3 µg of Cd [15], and the lungs absorb Cd more readily than the gastrointestinal tract [20,21]. The half-life of Cd is 20–30 years, and sustained Cd exposure increases the risk of lung diseases, such as lung cancer and COPD [20,21]. The Cd concentration observed in the lungs of smokers (30–50 µM) [22,23] reduces the CFTR expression and function [15,24]. 

Pseudomonas aeruginosa (PA) is an opportunistic bacterium that mainly infects immunocompromised individuals, including CF and COPD patients, contributing to the exacerbation of their symptoms [25,26]. Pyocyanin (PYO), a typical toxin secreted by PA [27], has been reported to be present in the sputum of CF patients in the range of 5–50 µM, and in some cases, 130 µM has been found [28]. PYO is converted to reduced PYO in cells to produce reactive oxygen species (ROS), which promotes inflammatory responses by facilitating the nuclear translocation of NF-κB [29]. Several studies have reported that PYO reduces the apical PM localization of CFTR by inhibiting vacuolar ATPase [30]. Moreover, PYO inhibits the cAMP-stimulated CFTR current [31]. Thus, sustained PYO exposure may be a deleterious factor inducing the acquired CFTR dysfunction, leading to severe exacerbation in COPD upon PA infection [32,33,34].

Previous studies have reported an increased incidence of the R75Q and M470V CFTR polymorphisms in COPD patients [35]. While the R75Q-CFTR channel has a normal Cl^−^ conductance, it has a selective defect in bicarbonate conductance, thereby increasing the risk of pancreatitis [36]. The M470V polymorphism has been reported to accelerate CFTR maturation; however, it reduces the channel open probability [37]. These CFTR polymorphisms associated with COPD may facilitate the acquired dysfunction of CFTR, especially upon sustained exposure to environmental pathogens, such as CS and bacterial infection. 

In this study, we investigated the effect of COPD-associated CFTR polymorphisms (R75Q, M470V) on the CFTR function in epithelial cell models in the presence of environmental pathogens and pollutants. We show that the R75Q or M470V polymorphism loses the CFTR function to suppress the proinflammatory cytokine production induced by COPD-related pathogens in the airway epithelial cells. In the presence of a COPD-related pathogen, the fractional PM activity (FPMA) of the CFTR Cl^−^ channel was dramatically reduced in airway epithelial cells expressing R75Q- or M470V-CFTR compared with WT-CFTR. Interestingly, we found that the CF drug Trikafta (VX-661, VX-445, and VX-770) improved the cell surface expression of R75Q- or M470V-CFTR CFTR and suppressed the excessive proinflammatory cytokine production. Thus, the pathogen-induced acquired CFTR dysfunction may be exaggerated in the COPD-associated CFTR variants, and the impaired suppression of excessive proinflammatory response to environmental pathogens may accelerate COPD pathogenesis.

## 2. Results

### 2.1. R75Q or M470V Polymorphism Loses CFTR’s Function to Suppress the Proinflammatory Cytokine Secretion

**Figure 1:**
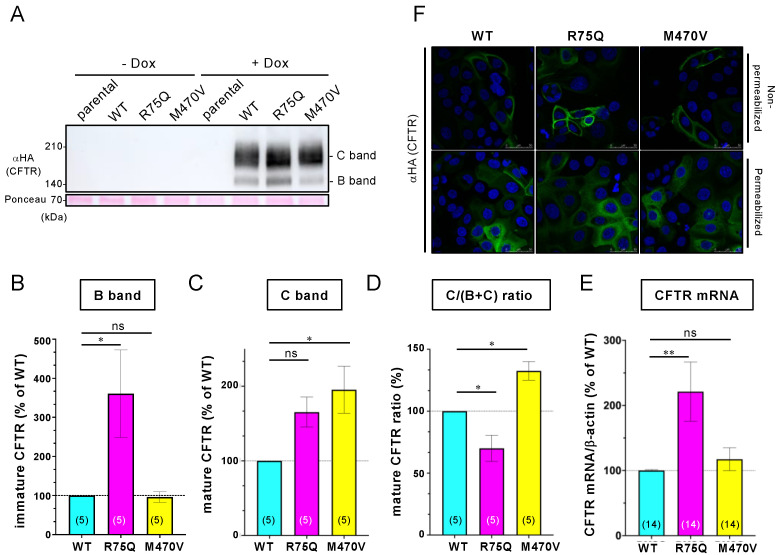
Establishment of CFBE Tet-on cells expressing WT, R75Q, or M470V-CFTR under a tetracycline-responsive promoter. (**A**) Western blotting using anti-HA antibody confirmed the expression of WT, R75Q, or M470V-CFTR with HBH tag at the N-terminus and 3HA tag in the extracellular region in CFBE Tet-on cells with (+Dox) or without 1 µg/mL dox treatment (-Dox) for 3 days. Ponceau staining was used as a loading control. B and C bands indicate immature and mature forms of CFTR, respectively. (**B**,**C**) Immature (B band, B) and mature (C band, C) forms of the CFTR variants were quantified by densitometry. (**D**) The C/(B+C) ratio was also quantified. (**E**) Exogenous CFTR variants’ mRNA levels in the CFBE Tet-on cells were quantified by qRT-PCR. (**F**) Fluorescence micrographs of CFBE Tet-on cells expressing WT, R75Q, or M470V-CFTR. Cellular localization of CFTR was analyzed by immunostaining using an anti-HA antibody with Alexa 488 conjugated secondary antibody in nonpermeabilized (upper) and permeabilized (lower) cells. DAPI was used for nuclear staining. Statistical significance was assessed by one-way ANOVA (* *p* < 0.05; ** *p* < 0.01; ns, not significant.) Data represent mean ± SE. The number of data from at least two independent experiments is indicated in parentheses.

Functional CFTR attenuates the proinflammatory cytokine secretion in the airway epithelial cells [38]. Several reports have shown that the R75Q or M470V polymorphism in CFTR is more prevalent in COPD patients than in normal individuals [35,39,40,41]. Thus, we investigated whether these CFTR polymorphisms suppress the proinflammatory response of airway epithelial cells to bacterial infection and CS exposure. To this end, we generated airway epithelial cells stably expressing R75Q- or M470V-CFTR as a model of COPD airway epithelial cells. We selected CFBE41o^−^ (CFBE) cells, well-characterized CF airway epithelial cells with no detectable CFTR expression [42]. We established CFBE Tet-on cells stably expressing WT-, R75Q-, or M470V-CFTR bearing an extracellular triple hemagglutinin (3HA) tag and N-terminal HBH tag [43] to induce the CFTR expression after doxycycline (Dox) treatment by lentiviral transduction. Western blotting showed that Dox treatment induced WT-, R75Q-, or M470V-CFTR as the immature and mature forms (Figure 1A). The densitometry analysis revealed that the expression level of immature R75Q-CFTR was significantly higher than that of WT-CFTR, while the mature form was comparable (Figure 1B,C). Maturation efficiency could be lower in R75Q-CFTR because the C/(B+C) ratio was significantly reduced compared with WT-CFTR (Figure 1D). The higher protein expression of R75Q-CFTR was likely due to the higher mRNA level of the exogenous R75Q-CFTR in the CFBE Tet-on cells (Figure 1E). In contrast, the expression level of the immature M470V-CFTR was comparable to WT; however, that of the mature form was significantly higher than that of WT-CFTR (Figure 1B,C). Maturation efficiency could be higher in M470V-CFTR because the ratio of the mature form to the total CFTR (C/(B+C) ratio) was significantly higher compared with WT-CFTR (Figure 1D). The higher maturation efficiency of M470V-CFTR may be due to the faster maturation as reported previously [37]. 

**Figure 2:**
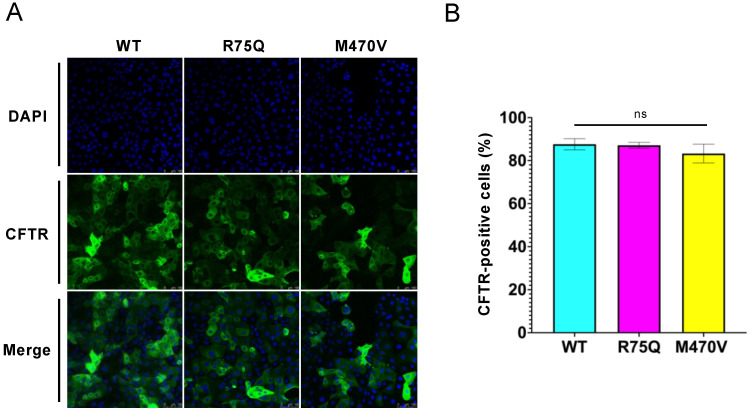
The transduction efficiency of the exogenous CFTR variants in the CFBE Tet-on cells. (**A**) Fluorescence micrographs of CFBE Tet-on cells expressing WT, R75Q, or M470V-CFTR immunostained using an anti-HA antibody with Alexa 488 conjugated secondary antibody in permeabilized cells. DAPI was used for nuclear staining. (**B**) The transduction efficiency was calculated as the percentage of the CFTR-expressing cells in 4–5 randomly selected areas containing >130 cells. The number of the cells used for the analysis is as follows; WT-CFTR 1402 cells, R75Q-CFTR 1280 cells, and M470V-CFTR 796 cells. Statistical significance was assessed by one-way ANOVA (ns, not significant). Data represent mean ± SE.

Immunofluorescence staining revealed that more than 80% of the cells expressed the exogenous WT-, R75Q-, or M470V-CFTR in the CFBE Tet-on cells we established (Figure 2A,B). Because the transduction efficiency of the exogenous CFTR was comparable between WT and variants (Figure 2B), the higher R75Q-CFTR mRNA level in the CFBE Tet-on cells may be from the variable numbers of integrated exogenous CFTR at high levels [38]. The variable numbers of integrated exogenous CFTR at high levels in the transduced CFBE cells have been reported [38,44]. Immunofluorescence staining of cell surface CFTR using its extracellular 3HA tags also revealed that similar to WT, R75Q- or M470V-CFTR can express at the PM (Figure 1F). Intracellular CFTR localization was also comparable between WT and variants (Figure 1F).

**Figure 3:**
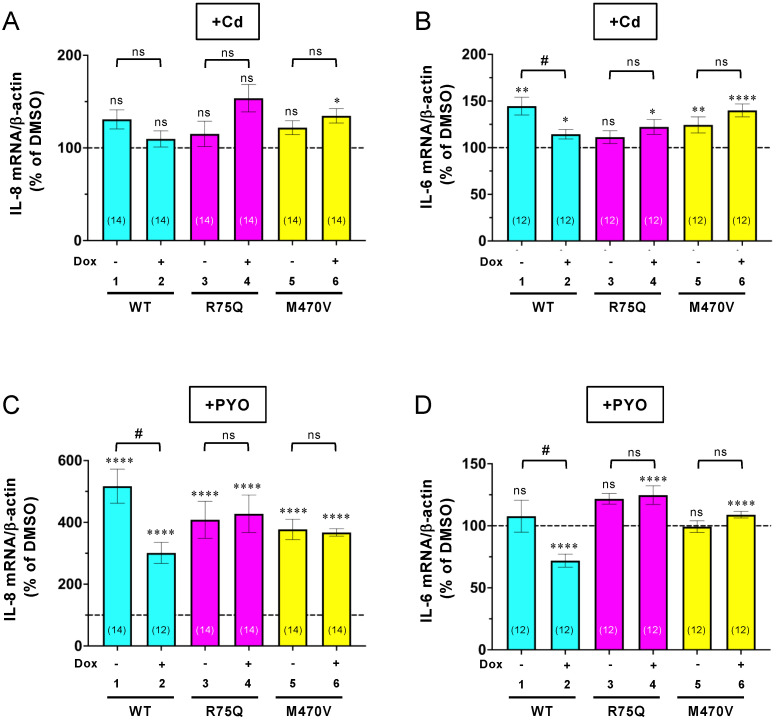
WT-CFTR, but neither R75Q nor M470V-CFTR, suppressed the Cd- or PYO-induced proinflammatory cytokine mRNA expression in CFBE Tet-on cells. CFBE Tet-on cells expressing WT-, R75Q-, or M470V-CFTR were treated with 15 µM Cd (**A**,**B**) and 5 µM PYO (**C**,**D**) for 3 days. At the same time, the cells were treated with or without Dox (1 µg/mL) for 3 days. Cd or PYO-induced mRNA expression of IL-8 was analyzed by RT-qPCR. Data represent mean±SE. n.s., not significant; * *p* < 0.05, ** *p* < 0.01, **** *p* < 0.0001 vs. each DMSO (*t*-test). # *p* < 0.05 vs. no Dox (*t*-test). The number of data from at least two independent experiments is indicated in parentheses.

**Figure 4:**
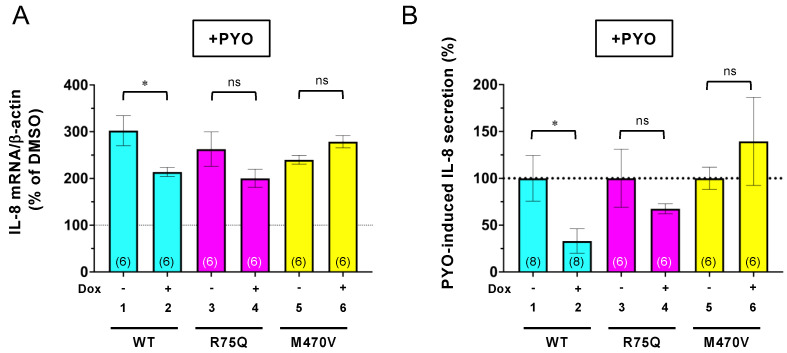
WT-CFTR, but neither R75Q nor M470V-CFTR, suppressed the PYO-induced IL-8 secretion in CFBE Tet-on cells. CFBE Tet-on cells expressing WT-, R75Q-, or M470V-CFTR were treated with or without Dox (1 µg/mL) for 3 days. PYO (5 µM) was treated for 24 h before analysis. (**A**) PYO-induced IL-8 mRNA expression was quantified by RT-qPCR (*n* = 6). (**B**) PYO-induced IL-8 secretion was analyzed by ELISA (*n* = 6). Data represent mean±SE. n.s., not significant; * *p* < 0.05 (*t*-test). The number of data from at least two independent experiments is indicated in parentheses.

Next, we investigated whether these COPD-associated CFTR polymorphisms affect the CFTR function in the inflammatory responses. As COPD-associated stimuli, we used Cd and PYO as the following regions. Cd, a main constituent of tobacco, induces CFTR dysfunction like CS [15]. PYO is a typical toxin secreted by PA whose infection seems to associate with symptoms of exacerbation in COPD [45,46,47,48]. Both Cd and PYO generate ROS [31], which are also violently released from alveolar macrophages in cigarette smokers with and without COPD [7]. Thus, to mimic a sustained oxidant burden as is present in cigarette smokers, we treated the CFBE cells with 15 µM Cd or 5 µM PYO for 3 days. Quantitative PCR (qPCR) assay showed that sustained Cd exposure induced IL-6 expression, but not IL-8 in CFBE Tet-on WT-CFTR cells without Dox treatment (Figure 3A,B, lane 1). In contrast, chronic PYO exposure dramatically induced IL-8, but not IL-6 in CFBE Tet-on WT-CFTR cells without Dox treatment (Figure 3C,D, lane 1). The WT-CFTR expression induced by Dox treatment reduced the Cd-induced IL-6 mRNA (Figure 3B, lanes 1–2) and the PYO-induced IL-8 mRNA (Figure 3C, lanes 1–2). In contrast, the R75Q- or M470V-CFTR expression failed to reduce the Cd-induced IL-6 mRNA (Figure 3B, lanes 3–6) and the PYO-induced IL-8 mRNA (Figure 3C, lanes 3–6). Similar results were obtained even after 24 h of treatment of the COPD-associated stimulus. While the WT-CFTR expression reduced the PYO-induced IL-8 mRNA (Figure 4A, lanes 1–2), the R75Q- or M470V-CFTR expression failed (Figure 4A, lanes 3–6). ELISA also showed that consistent with the mRNA level, the PYO-induced IL-8 secretion was also reduced by the expression of WT-CFTR (Figure 4B, lanes 1–2). However, the expression of R75Q- or M470V-CFTR failed to reduce the PYO-induced IL-8 secretion (Figure 4B, lanes 3–6). Despite that it is not a significant effect, the induced R75Q-CFTR expression tended to be slightly suppressed by the PYO-induced IL-8 production (Figure 4A,B). This may be due to the higher R75Q-CFTR protein expression compared with WT or M470V-CFTR (Figure 1A–C). These results suggest that the R75Q- or M470-CFTR variant loses CFTR’s function to suppress the inflammatory responses upon sustained exposure to the COPD-associated pathogen and pollutant.

### 2.2. Sustained Exposure to Cd or PYO Reduces the PM Level of R75Q-CFTR and M470V-CFTR Proteins to the Same Extent as WT-CFTR

**Figure 5:**
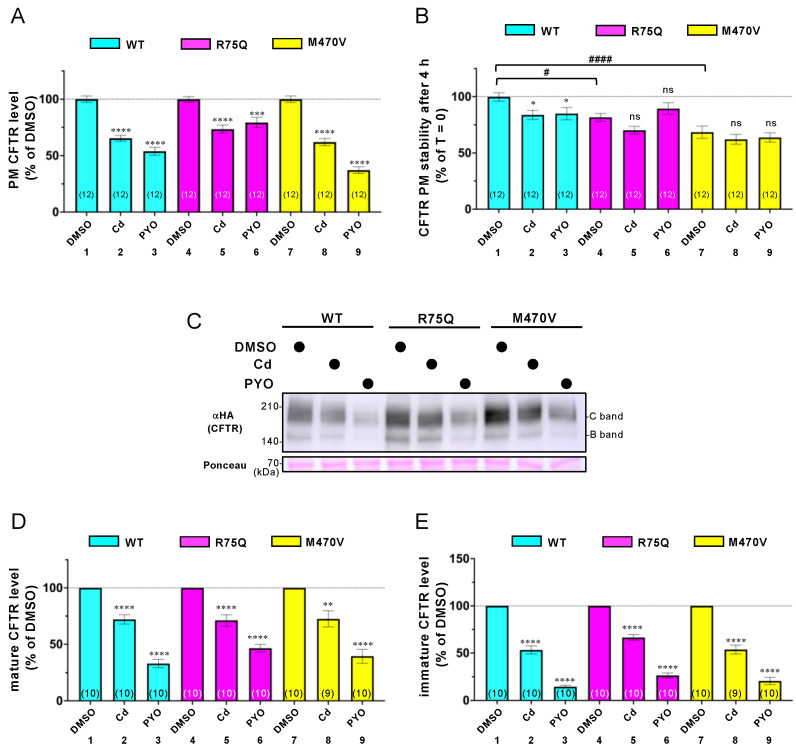
Effect of Cd or PYO on the PM density, stability, and expression of the CFTR variants. CFBE Tet-on cells expressing WT-, R75Q-, or M470V-CFTR were treated with 15 µM Cd or 5 µM PYO for 3 days in the presence of Dox (1 µg/mL). PM density (**A**) and PM stability (**B**) of CFTR were measured by cell surface ELISA using an anti-HA antibody. For the PM stability measurement, Cd or PYO was also treated during 4 h of incubation at 37 °C following anti-HA antibody binding. (**C**–**E**) The protein level of CFTR variants was measured by Western blotting. Ponceau staining was used as a loading control. B and C bands indicate immature and mature forms of CFTR, respectively. The mature (**D**) and immature (**E**) forms of CFTR were quantified by densitometry. Data represent mean±SE. The number of data from at least two independent experiments is indicated in parentheses. n.s., not significant; * *p* < 0.05, ** *p* < 0.01, *** *p* < 0.001, **** *p* < 0.0001 vs. DMSO (one-way ANOVA). # *p* < 0.05, #### *p* < 0.0001 vs. WT-CFTR (one-way ANOVA).

To clarify how the COPD-associated polymorphism loses the CFTR function, we examined its impact on the PM level in the CFBE cells. Cell surface ELISA using an anti-HA antibody showed that sustained exposure to Cd or PYO reduced the PM level of WT-CFTR in the CFBE Tet-on cells (Figure 5A, lanes 1–3). This phenotype is similar to the previous findings [15,24]. Unexpectedly, Cd or PYO exposure also reduced the PM level of R75Q- or M470V-CFTR to the same extent as WT-CFTR (Figure 5A, lanes 4–9). We also measured the PM stability of WT, R75Q-, or M470V-CFTR upon Cd or PYO exposure in the CFBE Tet-on cells. Cell surface ELISA revealed that R75Q- or M470V-CFTR was significantly unstable compared with WT-CFTR in the absence of COPD-associated pathogens (Figure 5B). Cd or PYO exposure slightly but significantly reduced the PM stability of WT-CFTR (Figure 5B), presumably by facilitating the internalization as CS induces [49]. In contrast, Cd or PYO exposure failed to reduce the PM stability of R75Q- or M470V-CFTR (Figure 5B). Thus, the reduced PM level of R75Q- or M470V-CFTR after sustained Cd or PYO exposure is unlikely due to the facilitated CFTR elimination from the PM. Rather, Cd or PYO exposure may interfere with the biosynthesis of R75Q- or M470V-CFTR, thereby reducing the cell surface level. 

We further performed Western blotting analysis to examine the effect of sustained exposure to Cd or PYO on the CFTR protein level in the CFBE Tet-on cells. Consistent with the effects on the CFTR PM level, sustained exposure to Cd or PYO reduced the mature and immature forms of R75Q- or M470V-CFTR to the same extent as WT-CFTR (Figure 5C–E). These results suggest that the functional defect of R75Q- or M470V-CFTR upon COPD-associated stimuli was unlikely due to the defective PM expression of the CFTR protein. 

### 2.3. Chronic PYO Exposure Inactivates the R75Q-CFTR Channel

**Figure 6:**
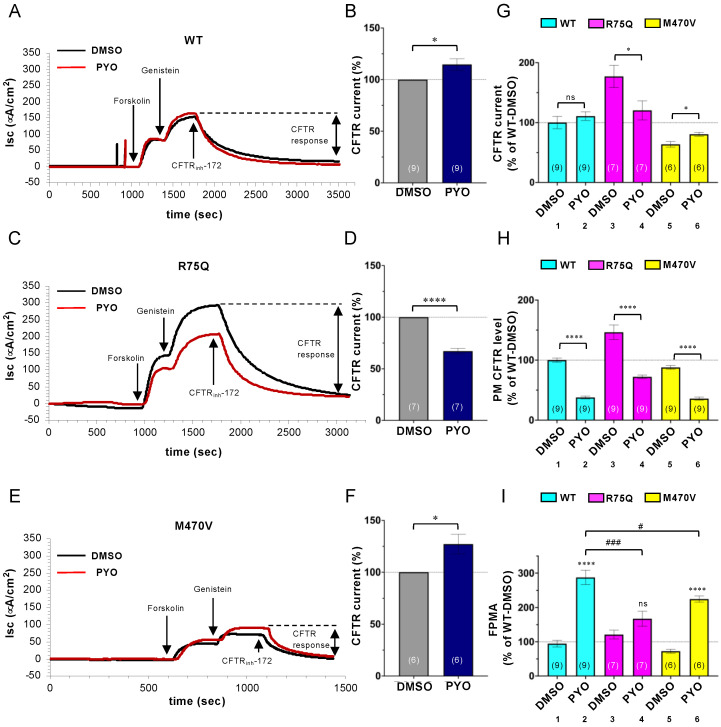
Effect of PYO on the Cl^−^ channel function of WT-, R75Q- or M470V-CFTR. (**A**–**F**) Apical CFTR current (Isc) in CFBE Tet-on cells expressing WT- (**A**,**B**), R75Q- (**C**,**D**), or M470V-CFTR (**E**,**F**) treated with Dox (50 ng/mL) for 3 days was measured after sequential addition of 10 μM forskolin and 100 μM genistein, followed by CFTR inhibition with 20 μM CFTRinh-172. PYO (5 µM) was treated for 2 days before Isc measurement. The effect of PYO on the WT- (**B**), R75Q- (**D**), and M470V-CFTR (**F**) Isc was calculated and expressed as % of the Isc in each DMSO-treated cell. (**G**) The Isc of CFTR variants was compared and expressed as % of the WT-CFTR Isc in the DMSO-treated cells. (**H**) The PM density of CFTR variants in CFBE Tet-on cells treated under the same condition as in G, except for Dox (1 µg/mL), was measured by cell surface ELISA. (**I**) The FPMA of CFTR variants was calculated as the ratio of the Isc (**G**) to PM density (**H**) and normalized to WT-CFTR in the DMSO-treated cells. Data represent mean±SE. The number of data from at least two independent experiments is indicated in parentheses. n.s., not significant; * *p* < 0.05, **** *p* < 0.0001 (*t*-test). # *p* < 0.05, ### *p* < 0.001 (one-way ANOVA).

Next, we examined the impact of COPD-associated polymorphism on the CFTR channel function. The polarized CFBE Tet-on cells were treated with 5 µM PYO for 2 days, and the CFTR activity was determined by short-circuit current measurement (Isc). Unlike the impact on the PM density, sustained PYO exposure slightly increased the CFTR current in CFBE Tet-on cells expressing WT-CFTR (Figure 6A,B). In contrast, PYO exposure significantly reduced the R75Q-CFTR current (Figure 6C,D). Similar to WT-CFTR, PYO exposure slightly increased the M470V-CFTR current (Figure 6E,F). The comparison of the CFTR current in the tested variants revealed that the R75Q-CFTR current was two times higher than WT-CFTR in the absence of PYO (Figure 6G). However, this was due to the higher PM density of R75Q-CFTR in the CFBE Tet-on cells (Figure 6H). We estimated the CFTR activation at the single-channel level by normalizing the CFTR current with the channel PM density measured by PM ELISA (Figure 6I), a readout designated as fractional PM activity (FPMA) [50]. The FPMA of WT-CFTR and R75Q-CFTR was comparable in the absence of PYO treatment; however, that of M470V-CFTR was ~70% of WT (Figure 6I), indicating that the M470V polymorphism reduces the CFTR channel gating as reported previously [37]. The PYO treatment increased the FPMA of WT-CFTR (Figure 6I), presumably as a host defense mechanism to increase fluid secretion [51]. In contrast, upon sustained PYO exposure, the FPMA of R75Q- or M470V-CFTR was significantly reduced compared with that of WT-CFTR (Figure 6I, lanes 2, 4, and 6). These results suggest that sustained exposure to environmental pathogens, such as PYO, may reduce the CFTR channel activity in individuals harboring R75Q- or M470V-CFTR polymorphism compared with individuals with WT-CFTR. 

### 2.4. Trikafta Corrects the Cell Surface Expression of CFTR upon PYO Exposure

**Figure 7:**
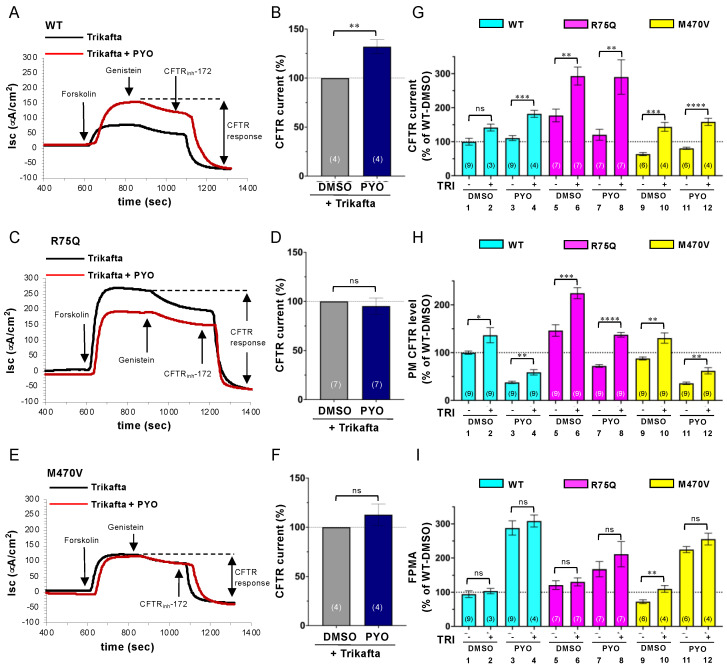
Trikafta corrects the PM expression and function of CFTR variants upon PYO exposure. (**A**–**F**) Apical CFTR current (Isc) in CFBE Tet-on cells expressing WT- (**A**, **B**), R75Q- (**C**, **D**), or M470V-CFTR (**E**, **F**) treated with Dox (50 ng/mL) for 3 days was measured as Figure 6. PYO (5 µM) was treated for 2 days before Isc measurement. Trikafta (3 µM VX-661, 3 µM VX-445, and 1 µM VX-770) was treated for 24 h before Isc measurement. The effect of PYO on the WT- (**B**), R75Q- (**D**), and M470V-CFTR (**F**) Isc upon Trikafta treatment was calculated and expressed as % of the Isc in each DMSO-treated cell. (**G**) The Isc of CFTR variants was compared and expressed as % of the WT-CFTR Isc in the DMSO-treated cells. The data in the cells without Trikafta (TRI) treatment shown in Figure 6G were used for comparison (odd lanes). (**H**) The PM density of CFTR variants in CFBE Tet-on cells treated under the same condition as in G, except for Dox (1 µg/mL), was measured by cell surface ELISA. (**I**) The FPMA of CFTR variants was calculated as the ratio of the Isc (**G**) to PM density (**H**) and normalized to WT-CFTR in the DMSO-treated cells. Data represent mean±SE. The number of data from at least two independent experiments is indicated in parentheses. n.s., not significant; * *p* < 0.05, ** *p* < 0.01, *** *p* < 0.001, **** *p* < 0.0001 (*t*-test).

Trikafta, a triple-combination drug composed of two CFTR correctors (tezacaftor/VX-661, elexacaftor/VX-445) and one CFTR potentiator (ivacaftor/VX-770), has been therapeutically used for CF with ∆F508-CFTR as it restores PM expression and function of CFTR with structural abnormalities [52,53]. Therefore, we examined whether Trikafta can ameliorate the PYO-induced CFTR dysfunction in the CFBE Tet-on cells. Even upon Trikafta treatment (3 µM VX-661, 3 µM VX-445, and 1 µM VX-770), PYO increased the WT-CFTR CFTR current similar to the nontreated cells (Figure 7A,B). Interestingly, while PYO reduced the R75Q-CFTR current in nontreated cells (Figure 6C,D), it failed to reduce the R75Q-CFTR current upon Trikafta treatment, indicating that Trikafta may prevent the PYO-induced R75Q-CFTR channel inactivation (Figure 7C,D). Unlike in the case of the nontreated cells (Figure 6E,F), PYO had no effect on the M470V-CFTR current upon Trikafta treatment (Figure 7E,F). Comparison analysis of the CFTR currents revealed that while Trikafta treatment failed to increase the WT-CFTR current in the absence of PYO exposure, it significantly increased the R75Q-CFTR and M470-CFTR current (Figure 7G). In contrast, Trikafta treatment increased the WT-CFTR, R75Q-CFTR, and M470-CFTR current upon PYO exposure (Figure 7G). PM ELISA also showed that Trikafta significantly increased the cell surface WT-CFTR, R75Q-CFTR, and M470V-CFTR in both nontreated and PYO-treated cells (Figure 7H). The FPMA of the CFTR variants was mostly unaffected by Trikafta treatment except for M470V-CFTR in the absence of PYO exposure (Figure 7I). These results indicate that Trikafta facilitates the cell surface appearance of WT-CFTR, R75Q-CFTR, and M470V-CFTR even in the presence of PYO exposure, leading to the increased Cl^−^ permeability at the apical PM of airway epithelial cells. 

### 2.5. Trikafta Corrects the Function of R75Q- or M470V-CFTR to Suppress the PYO-induced IL-8 Production

**Figure 8:**
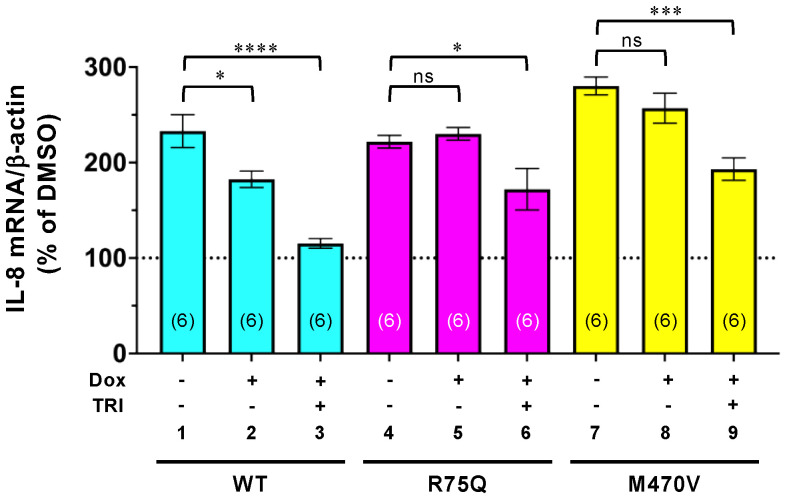
Trikafta suppresses the PYO-induced IL-8 expression in CFBE Tet-on cells. CFBE Tet-on cells expressing WT-, R75Q, or M470V-CFTR were treated with or without Dox (1 µg/mL) for 3 days. PYO (5 µM) and Trikafta (3 µM VX-661, 3 µM VX-445, and 1 µM VX-770) were treated for 24 h before analysis. PYO-induced IL-8 mRNA expression was quantified by RT-qPCR. Data represent mean±SE. The number of data from at least two independent experiments is indicated in parentheses. n.s., not significant; * *p* < 0.05, *** *p* < 0.001, **** *p* < 0.0001 vs. nontreated cells (one-way ANOVA).

Finally, we tested whether Trikafta suppresses the proinflammatory responses associated with PYO exposure. The proinflammatory cytokine IL-8 production in the CFBE Tet-on cells was quantified by qRT-PCR analysis after Trikafta and PYO treatment. While WT-CFTR expression reduced the PYO-induced IL-8 production, the IL-8 suppression effect was further enhanced by Trikafta treatment (Figure 8, lanes 1–3). The expression of neither R75Q-CFTR nor M470V-CFTR suppressed the PYO-induced IL-8 production (Figure 8, lanes 4–5 and lanes 7–8). However, upon Trikafta treatment, the expression of R75Q-CFTR or M470V-CFTR suppressed the PYO-induced IL-8 production (Figure 8, lanes 4–9). These results strongly suggest that Trikafta may suppress the proinflammatory responses associated with PYO exposure in airway epithelia harboring R75Q- or M470V-CFTR polymorphism.

## 3. Discussion

**Figure 9:**
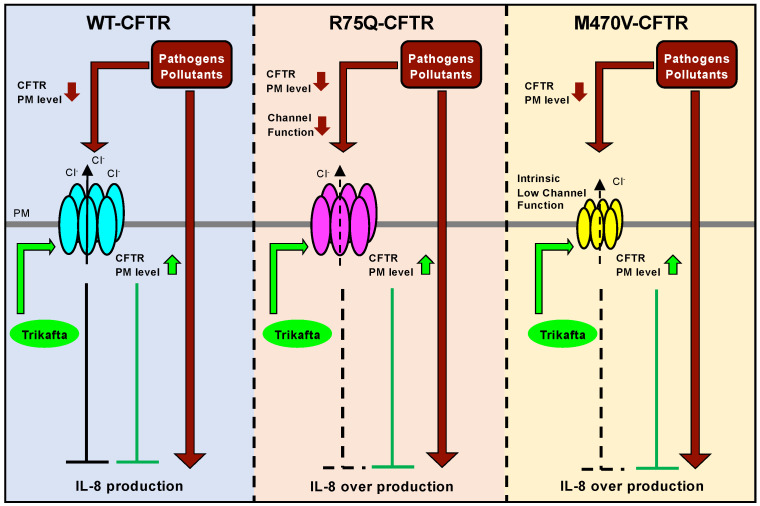
Putative mechanisms of the dysfunction of CFTR variants on the COPD-associated proinflammatory response. In normal airway epithelial cells expressing WT-CFTR, the production of proinflammatory cytokines, such as IL-8 and IL-6, is properly regulated because WT-CFTR suppresses excess cytokine production. Environmental pathogens and/or pollutants reduce the CFTR PM expression, leading to augmenting IL-8 production as a proinflammatory response. In the airway epithelial cells expressing R75Q-CFTR or M470V-CFTR, exposure to environmental pathogens and/or pollutants induces IL-8 overproduction because these CFTR variants fail to suppress cytokine production. Environmental pathogens and/or pollutants reduce the PM level and function of CFTR variants, which may further augment the dysfunction of CFTR variants frequently observed in COPD patients. CFTR modulator Trikafta can suppress the overproduction of proinflammatory cytokines in airway epithelial cells by increasing the PM expression of WT-CFTR and CFTR variants.

The present results demonstrate that WT-CFTR can suppress the proinflammatory cytokine production induced by COPD-associated pathogens and pollutants (e.g., Cd and PYO). On the other hand, the COPD-associated CFTR variant R75Q-CFTR or M470V-CFTR loses the ability to suppress the proinflammatory cytokine production. These results suggest a possibility that individuals carrying R75Q- or M470V-CFTR polymorphism are more susceptible to environmental pathogens and pollutants to induce excessive inflammatory responses, which may consequently accelerate COPD pathogenesis (Figure 9). Our results are consistent with previous findings that WT-CFTR suppresses the expression of inflammatory mediators [38]. Normally, CFTR contributes to the suppression of NF-κB during oxidative stress response by inhibiting the degradation of the B cell inhibitory factor (IκB) [54]. However, CF patients with CFTR dysfunction lack this response [54] and have increased inflammatory mediators, such as IL-8 and IL-6, leading to chronic inflammation [55,56]. In addition, CFTR participates not only in the transport of Cl^−^ and HCO_3_^−^, but also in the transport of GSH, an antioxidant and immune signaling molecule [57,58]. The inhibition of the transport of GSH to the airway surface fluid contributes to a decrease in the antioxidant capacity of epithelial cells and activates NF-κB [59,60,61,62,63]. Therefore, functional CFTR in airway epithelium is essential for the ability to transport Cl^−^, HCO_3_^−^, and GSH and to regulate the proper inflammatory responses against environmental pathogens and pollutants. 

It has been reported that R75Q-CFTR has a selective defect in HCO_3_^−^ conductance despite its functions normally as a Cl^−^ channel [36,41,64]. Consistent with previous studies, we showed that the FPMA of the R75Q-CFTR Cl^−^ channel was comparable to that of WT-CFTR in normal conditions. While PYO exposure reduced the PM density of the WT-CFTR channel, it increased the CFTR currents. Consequently, PYO enhanced the FPMA of the WT-CFTR Cl^−^ channel. The transient CFTR activation by CS has been reported in airway epithelia and is proposed to help protect the airway surface by diluting toxicants and facilitating their clearance by mucociliary transport [65]. Although the HCO_3_^−^ transport capacity of R75Q-CFTR was not evaluated in this study, we found that the FPMA of the R75Q-CFTR Cl^−^ channel was reduced to ~57% of the WT level upon PYO exposure. This reduced FPMA of R75Q-CFTR probably may result in the loss of CFTR function to suppress the excessive proinflammatory response. The reduced CFTR function upon pathogen exposure could also lead to reducing fluid secretion and impaired mucociliary clearance in airway epithelia, exaggerating the unregulated inflammatory responses. In support of this hypothesis, G551D-CFTR, a mutant defective in channel function, has been reported to lose the CFTR function to suppress the IL-8 secretion [38]. The mechanism of how PYO reduced the FPMA of R75Q-CFTR remains unclear. R75Q polymorphism may make CFTR more sensitive to channel oxidation compared with WT-CFTR as the oxidation state affects the CFTR channel gating kinetics [66,67]. It has been reported that oxidized CFTR channels have a slower opening rate, and consequently, the closed times are dramatically lengthened [66]. 

A common CFTR polymorphism, M470V, is found in individuals not affected by CF [37]. M470V-CFTR has been reported to be matured faster; however, it has only ~50% of intrinsic Cl^−^ channel activity compared with WT-CFTR [37]. Similar to this previous study, we found that the FPMA of the M470V-CFTR Cl^−^ channel was ~70% of WT-CFTR in the absence of COPD-associated pathogens. The intrinsic low activity of the M470V-CFTR Cl^−^ channel may be partially due to the conformational defect as we observed that the PM stability of M470V-CFTR was significantly lower than that of WT-CFTR, although the maturation efficiency could be higher than WT. Similar to WT-CFTR, the FPMA of the M470V-CFTR Cl^−^ channel was increased by PYO treatment. However, it was still significantly lower than the WT-CFTR level and may be insufficient for the CFTR function to suppress the proinflammatory responses. We cannot rule out another possibility that M470V-CFTR affects the HCO_3_^−^ and GSH transport, especially in the presence of environmental pathogens and pollutants. Future studies are needed to clarify the more precise mechanism.

CFTR modulators have been considered to apply therapeutic approaches for COPD [16,17,68]. The CFTR potentiator VX-770 is shown to reverse the changes made by CS in the human bronchial epithelium by increasing the CFTR channel function, restoring the cell surface fluid, and improving mucociliary clearance [69,70,71]. In this study, we provide evidence that Trikafta may correct the acquired CFTR dysfunction induced by environmental pathogens. We showed that R75Q- or M470V-CFTR was significantly unstable at the PM of the CFBE cells compared with WT-CFTR, indicating the intrinsic conformational instability of R75Q- or M470V-CFTR because the PM stability is well correlated with the conformational stability [72]. The lower maturation efficiency of R75Q-CFTR may also reflect conformational instability. We found that Cd or PYO exposure significantly reduced the PM stability of WT-CFTR. These results suggest that environmental pathogens and pollutants may induce CFTR conformational instability. Consistent with this hypothesis, it has been reported that CS induces acute misfolding of cell surface CFTR and internalization, ultimately forming intracytoplasmic aggregates in the epithelial cells [13,49]. Moreover, a previous study showed that the corrector-rescued ∆F508-CFTR, which is conformationally less stable compared with WT-CFTR [72], was more susceptible to CS-induced channel inactivation [65]. Thus, environmental pathogens and pollutants may induce conformational CFTR instability, and R75Q or M470V polymorphism may further facilitate this acquired CFTR conformational instability, resulting in reduced PM expression. Trikafta may correct the acquired CFTR conformational instability induced by environmental pathogens and pollutants by directly binding to CFTR [73]. Consequently, Trikafta restored the PM expression of R75Q- or M470V-CFTR even in the presence of environmental pathogens and pollutants, thereby suppressing the excess inflammatory response, which may lead to COPD pathogenesis (Figure 9).

In summary, we provide a possible mechanism of how the CFTR polymorphism, especially R75Q- or M470V-CFTR, facilitates proinflammatory responses, which could associate with COPD pathogenesis in an airway epithelial cell model. This study also provides evidence that the CFTR modulator Trikafta could counteract the acquired CFTR dysfunction even in R75Q- or M470V-CFTR; thus, it may be applicable to a therapeutic approach to prevent COPD pathogenesis.

## 4. Materials and Methods

### 4.1. Constructs

His-Biotin-His (HBH)-R75Q-CFTR harboring an extracellular 3HA tag at the 4th extracellular loop (HBH-R75Q-CFTR-3HA) or HBH-M470V-CFTR-3HA was constructed in the pDONR221 vector (Thermo Fisher) by PCR mutagenesis using HBH-WT-CFTR-3HA [43] as a template. Inducible lentiviral expression plasmids of HBH-WT, R75Q-, or M470V-CFTR-3HA were constructed in pLIX402 (Addgene #41394) by LR reaction using LR clonase II (Thermo Fisher, Waltham, MA, USA). Detailed information is available from the authors on request.

### 4.2. Cell Culture and Reagents

The human CF bronchial epithelial cell line CFBE41o- (CFBE), with a CFTR∆F508/∆F508 genotype, was obtained from D. Gruenert (University of California, San Francisco) and grown as reported previously [43]. CFBE Tet-on cells stably expressing HBH-WT-CFTR-3HA, HBH-R75Q-CFTR-3HA, or HBH-M470V-CFTR-3HA under a tetracycline-responsive promoter were generated by lentivirus transduction under puromycin (3 µg/mL) selection as reported previously [43]. Briefly, lentiviral particles expressing HBH-WT-, M470V-, or M470V-CFTR-3HA were produced in the HEK293T cells with the Lenti-X Packaging System (Takara Bio, Japan) following the manufacturer’s instructions. The cell lines were generated by transduction with lentiviral particles containing the inducible HBH-CFTR-3HA, followed by selection with puromycin for 2 weeks.

Cadmium nitrate tetrahydrate (Cd), pyocyanin (PYO), and doxycycline hydrochloride (Dox) were purchased from FUJIFILM Wako Chemicals (Japan). VX-809, VX-661, and VX-770 were purchased from AdooQ BioScience (Irvine, CA, USA). VX-445 and DMSO were purchased from Selleck Chemicals (Houston, TX, USA) and Sigma-Aldrich (St. Louis, MO, USA), respectively. 

### 4.3. Immunocytochemistry

The subcellular localization of the HBH-CFTR-3HA variant in CFBE Tet-on cells was examined as reported previously [74]. The cells, plated on a coverslip, were washed twice with a phosphate-buffered saline (PBS) and fixed with 4% paraformaldehyde (FUJIFILM-Wako Chemicals, Japan) for 30 min at room temperature (RT). Cells were permeabilized with 0.1% Triton X-100 in PBS for 5 min and blocked with 0.5% bovine serum albumin (BSA) in PBS for 20–30 min at RT. Cells were incubated for 2 h at RT with an anti-HA antibody (16B12, BioLegend, San Diego, CA, USA, 1:1000) in 0.5% BSA in PBS. Then, the cells were washed three times with PBS, and an Alexa Fluor^®^ 488–conjugated anti-mouse IgG (1:500, Jackson ImmunoResearch, West Grove, PA, USA) was added to the cells, which were then incubated for 1 hour at RT. After washing the cells three times with PBS, a DAPI solution (Peptide Institute, Inc., Japan) diluted in PBS (1:5000) was added to the cells, which were then incubated for 5 min at RT. After washing the cells three times with PBS, the cells were mounted with a Vectashield^®^ mounting medium (Vector Laboratories, Newark, CA, USA). The images were visualized and captured with an inverted laser confocal fluorescence microscope (SP8, Leica Microsystems GmbH, Germany). 

### 4.4. Quantitative Real-Time PCR 

Total RNA was extracted from CFBE cells grown on a 24-well plate using TRIzol^®^ (Thermo Fisher, Waltham, MA, USA) according to the manufacturer’s protocols. An amount of 500 ng of total RNA was then used for the reverse transcription reaction using ReverTra Ace^®^ qPCR RT Master Mix (Toyobo, Japan). Quantitative RT-PCR was performed in the LightCycler^®^ 480 System (Roche Diagnostics, Switzerland), and the gene expression was examined by SYBR Advantage qPCR Premix (Toyobo, Japan). The relative quantity of the target gene mRNA was normalized using human β-actin as the internal control and expressed as the relative quantity of the target gene mRNA (-fold induction). PCR amplification was performed in 2 steps, and the reaction protocol included preincubation at 95 °C for 3 min; then the amplification of 40 cycles was set for 5 s at 95 °C and 30 s at 60 °C, and the melting curve of 5 min at 95 °C, 60 s at 60 °C, and 97 °C. The sequences of primers used for quantitative RT-PCR are listed in Table 1.

### 4.5. IL-8 ELISA

The confluent CFBE Tet-on cells were treated with 1 µg/mL Dox for 2 days, followed by a 1-day treatment with 1 µg/mL Dox and 5 µM PYO for 24 h at 37 °C. After the PYO treatment, the culture medium was collected and used for ELISA after centrifugation at 12,000 rpm for 5 min. A fresh cell culture medium was used to measure the background signal. Human IL-8 was measured in cell culture media by the DuoSet^®^ Human IL-8/CXCL8 ELISA kit (R&D Systems, Minneapolis, MN, USA) according to the manufacturer’s instructions. PYO-induced IL-8 secretion was quantified by subtracting the IL-8 secretion in DMSO-treated cells from that in PYO-treated cells.

### 4.6. Cell Surface Density and Cell Surface Stability Measurements of CFTR

The PM density and stability of the HBH-CFTR-3HA variants in CFBE Tet-on cells on 24-well plates were measured by cell surface ELISA as reported previously [43]. Briefly, cell surface CFTR-3HA was labeled with anti-HA antibody (16B12, BioLegend, San Diego, CA, USA), biotinylated anti-mouse IgG (Funakoshi, Japan), and NeutrAvidin protein-HRP (Thermo Fisher, Waltham, MA, USA) and detected by the Amplex Red reagent (Thermo Fisher, Waltham, MA, USA). For the PM stability measurement, Cd or PYO was added to the cell culture medium during the 4 h’ chase at 37 °C incubation. 

### 4.7. Western Blotting

The CFBE cells were solubilized in RIPA buffer (150 mM NaCl, 50 mM Tris-HCl, 1% NP-40, 0.5% sodium deoxycholate, 0.1% SDS, and pH 8.0) supplemented with 1 mM PMSF, 5 µg/mL leupeptin, and pepstatin. Western blotting experiments were performed as reported previously [43]. After detecting the total proteins in the blotting membrane by Ponceau S (Sigma-Aldrich, St. Louis, MO, USA) staining, the HBH-CFTR-3HA variants were detected by an anti-HA antibody (16B12, BioLegend, San Diego, CA, USA) and an HRP-conjugated secondary antibody (Jackson ImmunoResearch, West Grove, PA, USA). The antigen–antibody complexes were incubated with a SuperSignal West Pico PLUS Chemiluminescent Substrate (Thermo Fisher, Waltham, MA, USA) and analyzed by the Fusion chemiluminescence imaging system (Vilber Bio Imaging, France).

### 4.8. Short-Circuit Current Measurement

CFBE Tet-on cells were plated on fibronectin-coated, 12 mm Snapwell filters (Corning, Corning, NY, USA) at a density of 1 × 10^5^ cells/cm^2^. After reaching confluence, cells were treated with Dox (50 ng/mL) for 3 days to induce the CFTR variants. The polarized epithelia (≥5 days after confluence) were mounted in Ussing chambers (U-2500, Warner Instruments, Holliston, MA, USA), bathed in Krebs-bicarbonate Ringer, and continuously bubbled with 95% O_2_ and 5% CO_2_. To impose a Cl^−^ gradient, Cl^−^ was replaced by gluconate in the apical compartment. To functionally isolate the apical membranes, the basolateral PM was permeabilized with 100 µM amphotericin B (FUJIFILM Wako Chemicals, Japan), and the epithelial sodium channel was inhibited with 100 μM amiloride (TCI, Japan). CFTR activity was stimulated by apical forskolin (10 µM, FUJIFILM Wako Chemicals) and genistein (100 µM, FUJIFILM Wako Chemicals, Japan), followed by the addition of CFTR inhibitor 172 (Inh172, 20 μM, Santa Cruz Biotechnology, Dallas, TX, USA) to determine CFTR-specific apical Cl- current. Measurements were performed at 37 °C and recorded using a current-clamp amplifier (CEZ-9100, Nihon Kohden, Japan) and PowerLab 2/26 system (ADInstruments, New Zealand).

### 4.9. Statistical Analysis

For quantification, replicate experiments were repeated at least two times, and data were expressed as means ± SE. Statistical significance was assessed by either a two-tailed paired Student’s *t*-test or a one-way ANOVA using GraphPad Prism 8 (GraphPad Software, Version 8.4.3).

## Figures and Tables

**Table 1:** Primers used for quantitative RT-PCR.

Primer	Orientation	Sequence
Human β-actin	Forward	5′-ACTCTTCCAGCCTTCCTTCC-3′
	Reverse	5′-GAGGAGCAATGATCTTGATCTTC-3′
Human IL-8	Forward	5′-TCCTGATTTCTGCAAGCTCTG-3′
	Reverse	5′-GTCCACTCTCAATCACTCTCAG-3′
Human IL-6	Forward	5′-GCACTGGCAGAAAACAACCT-3′
	Reverse	5′-CAGGGGTGGTTATTGCATCT-3′
Human CFTR	Forward	5′-AGTGGAGGAAAGCCTTTGGAGT-3′
	Reverse	5′-ACAGATCTGAGCCCAACCTCA-3′

## Data Availability

Not applicable.
